# Citicoline for traumatic brain injury: a systematic review & meta-analysis

**DOI:** 10.5249/jivr.v9i1.843

**Published:** 2017-01

**Authors:** Ali Meshkini, Mohammad Meshkini, Homayoun Sadeghi-Bazargani

**Affiliations:** ^*a*^Department of Neurosurgery, Neuroscience Research Center, Tabriz University of Medical Sciences, Tabriz, Iran.; ^*b*^Tabriz University of Medical Sciences (Bio-Medicine), Tabriz, Iran.; ^*c*^Road Traffic injury Research Center, Department of Statistics and Epidemiology, Tabriz University of Medical Sciences, Tabriz, Iran.

**Keywords:** Traumatic brain injury, Head injury, Neuroprotective agents, Citicoline, Glasgow outcome Scale, Review, Meta-analysis

## Abstract

**Background:**

Traumatic Brain Injury (TBI) is the leading cause of mortality and morbidity especially in young ages. Despite over 30 years of using Neuroprotective agents for TBI management, there is no absolute recommended agent for the condition yet.

**Methods:**

This study is a part of a scoping review thesis on "Neuroprotective agents using for Traumatic Brain Injury: a systematic review & meta-analyses", which had a wide proposal keywords and ran in "Cochrane CENTRAL", "MedLine/PubMed", "SCOPUS", "Thomson Reuters Web of Science", "SID.ir", "Barket Foundation", and "clinicaltrials.gov" databases up to September 06, 2015. This study limits the retrieved search results only to those which used \citicoline for TBI management. The included Randomized Clinical Trials’ (RCTs) were assessed for their quality of reporting by adapting CONSORT-checklist prior to extracting their data into meta-analysis. Meta-analyses of this review were conducted by Glasgow Outcome Scale (GOS) in acute TBI patients and total neuropsychological assessments in both acute and chronic TBI management, mortalities and adverse-effects.

**Results:**

Four RCTs were retrieved and included in this review with 1196 participants (10 were chronic TBI impaired patients); the analysis of 1128 patients for their favorable GOS outcomes in two studies showed no significant difference between the study groups; however, neuropsychological outcomes were significantly better in placebo/control group of 971 patients of three studies. Mortality rates and adverse-effects analysis based on two studies with 1429 patients showed no significant difference between the study groups. However, two other studies have neither mortality nor adverse effects reports due to their protocol.

**Conclusions:**

Citicoline use for acute TBI seems to have no field of support anymore, whereas it may have some benefits in improving the neuro-cognitive state in chronic TBI patients. It’s also recommended to keep in mind acute interventions like Psychological First Aid (PFA) during acute TBI management.

## Introduction

Traumatic Brain Injury (TBI), also known as Head Injury,^1-3^ is the leading cause of mortality and morbidity ^1,4-6^ and mostly affects young people.^1^ It has an incidence ratio of 558 new cases per 100,000 people each year with more than 50,000 mortalities, it’s also estimated to cause 33 new disabilities per 100,000 people in a year; and to cost more than $48 billion a year. About 2.5 to 6.5 million Americans have experienced a TBI event through their life.^1,4^ It is important to mention that "Survivors of TBI are often left with significant cognitive, behavioral, and communicative disabilities".^7^

Adenosine Tri-Phosphate (ATP) is responsible for Cell Membrane Sodium-Potassium(Na-K) ATPase Pump’s Function. TBI related cell membrane un-integrity and accumulation of extracellular water lead to the brain edema and formation of lipid peroxidase. Cholinergic agents (e.g. Citicoline) have effects on cell-oxygenation cycles and formation of ATP, which may indirectly rebuild cell wall integrity, reducing further secondary injuries.^8^

## Methods

**Study Design**

This is a systematic review and meta-analysis of RCTs.

**Search Strategy and Inclusion Criteria**

This study is a part of a scoping review thesis on "Neuroprotective agents using for traumatic brain injury: a systematic review & meta-analyses" whose search strategy was not restricted by date, race, gender, and publication status; however, date limitation to the reference databases (i.e. SCOPUS and Thomson Reuters Web of Science) was implemented for studies after 2000.

The "Cochrane CENTRAL", "Medline through PUBMED", "SCOPUS", "Thomson Reuters Web of Science", "SID.ir", "Barekat Knowledge Deployment Foundation (formerly known as IRAN-MEDEX)" and "clinicaltrials.gov" databases were searched by September 06, 2015 (appendices 1-7, present the full search strategies). Other related articles not retrieved from database search results were also included. These were related articles encountered during internet search process for full-text articles, full-text requests through www.researchgate.net; skimming bibliographies of articles and contacting experts in the field. Study’s PICO design could be summarized as following:

**Appendix 1:** Cochrane CENTRAL Search Strategy

	
Search Name:	Cochrane CENTRAL Search strategy
Date Run:	06/09/15 15:46:13.919
Description:	Cochrane CENTRAL Search strategy
ID	Search Hits
#1	traumatic brain injur*:ti,ab,kw (Word variations have been searched) 1394
#2	traumatic head injur*:ti,ab,kw 497
#3	brain injur*:ti,ab,kw 3417
#4	#1 or #2 or #3 3529
#5	Neuroprotect*:ti,ab,kw 1669
#6	Neuro-protect*:ti,ab,kw 17
#7	Piracetam:ti,ab,kw 594
#8	Neuroaid:ti,ab,kw 43
#9	citicoline:ti,ab,kw 151
#10	hyperventilation:ti,ab,kw 688
#11	hyperbaric oxygen:ti,ab,kw 852
#12	hyperbaric O2:ti,ab,kw 47
#13	#5 or #6 or #7 or #8 or #9 or #10 or #11 or #12 3934
#14	#4 and #13 291

**Appendices:** Appendices 2-7

**Appendix 2: Medline/PubMed CENTRAL Search Strategy** ((traumatic[All Fields] AND (brain injure[All Fields] OR brain injured[All Fields] OR brain injures[All Fields] OR brain injured[All Fields] OR brain injuries[All Fields] OR brain injury[All Fields] OR brain injury,[All Fields])) OR (traumatic[All Fields] AND (head injure[All Fields] OR head injured[All Fields] OR head injures[All Fields] OR head injured[All Fields] OR head injuries[All Fields] OR head inju-ry[All Fields])) OR (brain injure[All Fields] OR brain injured[All Fields] OR brain injures[All Fields] OR brain injured[All Fields] OR brain injuries[All Fields] OR brain injury[All Fields] OR brain injury,[All Fields])) AND ((neuroprotect[All Fields] OR neuroprotectans[All Fields] OR neuroprotectant[All Fields] OR neuroprotectant'[All Fields] OR neuroprotectant,[All Fields] OR neuroprotectants[All Fields] OR neu-roprotectective[All Fields] OR neuroprotected[All Fields] OR neuroprotecteur[All Fields] OR neuroprotecteurs[All Fields] OR neuropro-tectia[All Fields] OR neuroprotectice[All Fields] OR neuroprotectie[All Fields] OR neuroprotectief[All Fields] OR neuroprotectin[All Fields] OR neuroprotectind1[All Fields] OR neuroprotecting[All Fields] OR neuroprotectins[All Fields] OR neuroprotection[All Fields] OR neuroprotection'[All Fields] OR neuroprotection,[All Fields] OR neuroprotectionary[All Fields] OR neuroprotectionl[All Fields] OR neuro-protections[All Fields] OR neuroprotectionwith[All Fields] OR neuroprotectiove[All Fields] OR neuroprotective[All Fields] OR neuropro-tective'[All Fields] OR neuroprotectively[All Fields] OR neuroprotectiveness[All Fields] OR neuroprotectives[All Fields] OR neuroprotec-tivion[All Fields] OR neuroprotectivity[All Fields] OR neuroprotector[All Fields] OR neuroprotectora[All Fields] OR neuroprotectoras[All Fields] OR neuroprotectores[All Fields] OR neuroprotectors[All Fields] OR neuroprotectory[All Fields] OR neuroprotectrice[All Fields] OR neuroprotectrices[All Fields] OR neuroprotectrives[All Fields] OR neuroprotects[All Fields] OR neuroprotectve[All Fields]) OR (neuro protectant[All Fields] OR neuro protectants[All Fields] OR neuro protecting[All Fields] OR neuro protectins[All Fields] OR neuro protec-tion[All Fields] OR neuro protective[All Fields] OR neuro protector[All Fields]) OR ("piracetam"[MeSH Terms] OR "piracetam"[All Fields]) OR ("Neuroaid"[Supplementary Concept] OR "Neuroaid"[All Fields] OR "neuroaid"[All Fields]) OR ("cytidine diphosphate cho-line"[MeSH Terms] OR ("cytidine"[All Fields] AND "diphosphate"[All Fields] AND "choline"[All Fields]) OR "cytidine diphosphate cho-line"[All Fields] OR "citicoline"[All Fields]) OR ("hyperventilation"[MeSH Terms] OR "hyperventilation"[All Fields]) OR (hyperbaric[All Fields] AND ("oxygen"[MeSH Terms] OR "oxygen"[All Fields])) OR (hyperbaric[All Fields] AND O2[All Fields])) AND Clinical Trial[ptyp]

**Appendix 3: SCOPUS CENTRAL Search Strategy** (TITLE-ABS-KEY(traumatic brain injur*) OR TITLE-ABS-KEY(traumatic head injur*) OR TITLE-ABS-KEY(brain injur*)) AND ((TITLE-ABS-KEY(Neuroprotect*) OR TITLE-ABS-KEY(Neuro-protect*) OR TITLE-ABS-KEY(Piracetam) OR TITLE-ABS-KEY(Neuroaid) OR TITLE-ABS-KEY(citicoline) OR TITLE-ABS-KEY(hyperventilation) OR TITLE-ABS-KEY(hyperbaric oxygen) OR TITLE-ABS-KEY(hyperbaric O2)) AND (TITLE-ABS-KEY(RCT) OR TITLE-ABS-KEY(Trial*) OR TITLE-ABS-KEY(Random*)) AND NOT rat* AND ( LIMIT-TO(PUBYEAR,2015) OR LIMIT-TO(PUBYEAR,2014) OR LIMIT-TO(PUBYEAR,2013) OR LIMIT-TO(PUBYEAR,2012) OR LIMIT-TO(PUBYEAR,2011) OR LIMIT-TO(PUBYEAR,2010) OR LIMIT-TO(PUBYEAR,2009) OR LIMIT-TO(PUBYEAR,2008) OR LIMIT-TO(PUBYEAR,2007) OR LIMIT-TO(PUBYEAR,2006) OR LIMIT-TO(PUBYEAR,2005) OR LIMIT-TO(PUBYEAR,2004) OR LIMIT-TO(PUBYEAR,2003) OR LIMIT-TO(PUBYEAR,2002) OR LIMIT-TO(PUBYEAR,2001) OR LIMIT-TO(PUBYEAR,2000) )

**Appendix 4: Thomson Reuters Web of Science CENTRAL Search Strategy** TOPIC: (Brain Injury) OR TOPIC: (Brain Damage) OR TOPIC: (Traumatic Brain Injury) OR TOPIC: (Traumatic Head Injury) OR TOPIC: (Traumatic Brain Injuries) OR TOPIC: (Traumatic Head Injuries) OR TOPIC: (Brain Injuries) OR TOPIC: (Brain Damages) Refined by: TOPIC: (neuroprotec*) AND DOCUMENT TYPES: ( ARTICLE ) AND TOPIC: (Clinical Trials) Timespan: 2000-2015. Indexes: SCI-EXPANDED, SSCI, CPCI-S, CPCI-SSH.

**Appendix 5: SID.ir CENTRAL Search Strategy** Scientific Information Database "SID.ir" indexes Iranian authors information in 3 subgroups: Journals Articles, Non-Journal Arti-cles(Conference Articles, etc.) & Theses Proposals(unpublished works); of both English & Persian Languages. Our Search Strategy due to it's limited potency for defining search strategy, conducted to search through Titles, Abstracts & Keywords for related articles of "Brain Injury" or its Persian equivalents; and hand-search through the results for related Articles.

**Appendix6: Barekat (Iran MEDEX) CENTRAL Search Strategy** The most important difference between "SID.ir" &"Iran-MEDEX" is "Iran-MEDEX" indexes the articles of Islamic Scientific Citation(ISC) Journals, other than Iranian journals; the same search strategy of "SID.ir" belongs to this Database too.

**Appendix 7: clinicaltrials.gov CENTRAL Search Strategy** "Brain Injury" AND "Neuroprotective" | Interventional Studies (48 records)

• Patients of any age, and with any severity (mild, moderate, severe) of focal or diffuse, acute or chronic TBI. Animal studies or pre-clinical (in-vivo) trials were excluded from this study.

• Intervention (Specified to current study): any form and dosage of citicoline use;

• Compared to placebo/conventional treatment control group patients;

• Outcomes assessed as: (1) favorably outcome of intervention (good recovery and mild disability based on GOS or improvement in Neuropsychological state), (2) mortality and vegetative-state (based on GOS), (3) probable side-effects of citicoline.

**Review Method and Study-Selection**

**Figure 1:**
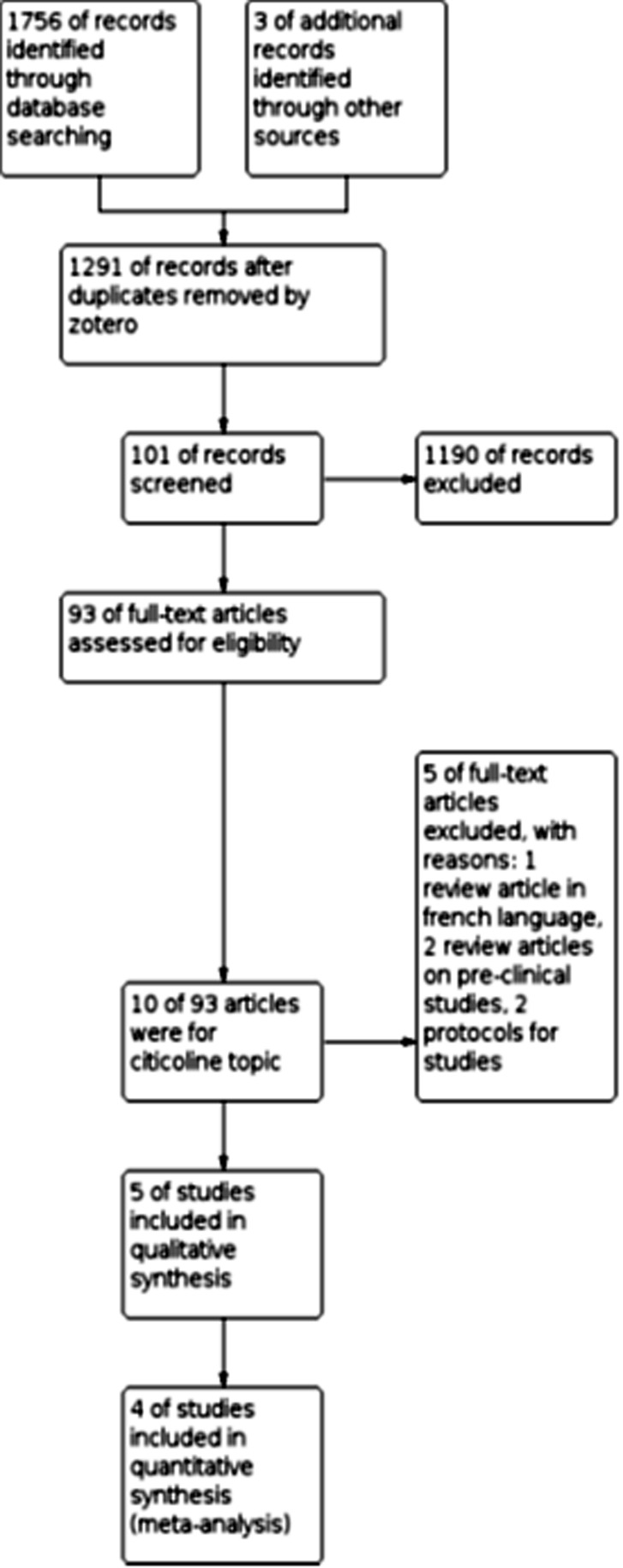
Study flow diagram (PRISMA Template)

After eliminating duplicates from search results with Zotero v.4.0.28 (available from www.zotero.org which was used as reference manager too), retrieved articles were screened via their titles and abstracts by two review authors. Further assessment of retrieved RCTs for their quality of reporting and eligibility to extract data for quantitative analysis was done by applying Consolidated Standards Of Reporting Trials (CONSORT-checklist) 2010 (available from http://www.consort-statement.org/) on full-texts of the articles by two review authors (appendix 8  demonstrates CONSORT 2010 Checklist). It was planned to refer to any disagreements in screening-phase and the decision on including studies to the third author. However, no such conflict was encountered in this review. There were also other reviews and protocol papers in search results that were excluded from the study process due to being a pre-clinical study or using non-English language. The flow of identification and selection of studies was reported using the Preferred Reporting Items for Systematic Reviews and Meta-Analyses (PRISMA) flowchart, which was modified for citicoline topic in current paper (Figure 1).

**Appendix 8:** CONSORT 2010 checklist of information to include when reporting a randomized trial*

Section/Topic	Item No	Checklist item	Reported on page No
**Title and abstract**		
	1a 1b	Identification as a randomized trial in the title Structured summary of trial design, methods, results, and conclusions (for specific guidance see CONSORT for abstracts)
**Introduction**		
Background and objectives	2a 2b	Scientific background and explanation of rationale Specific objectives or hypotheses
**Methods**		
Trial design	3a 3b	Description of trial design (such as parallel, factorial) including allocation ratio Important changes to methods after trial commencement (such as eligibility criteria), with reasons
Participants	4a 4b	Eligibility criteria for participants Settings and locations where the data were collected
Interventions	5	The interventions for each group with sufficient details to allow replication, including how and when they were actually admin-istered
Outcomes	6a 6b	Completely defined pre-specified primary and secondary outcome measures, including how and when they were assessed Any changes to trial outcomes after the trial commenced, with reasons
Sample size	7a 7b	How sample size was determined When applicable, explanation of any interim analyses and stopping guidelines
**Randomization:**		
Sequence generation	8a 8b	Method used to generate the random allocation sequence Type of randomization; details of any restriction (such as blocking and block size)
Allocation concealment mechanism	9	Mechanism used to implement the random allocation sequence (such as sequentially numbered containers), describing any steps taken to conceal the sequence until interventions were assigned
Implementation	10	Who generated the random allocation sequence, who enrolled participants, and who assigned participants to interventions
Blinding	11a 11b	If done, who was blinded after assignment to interventions (for example, participants, care providers, those assessing outcomes) and how If relevant, description of the similarity of interventions
Statistical methods	12a 12b	Statistical methods used to compare groups for primary and secondary outcomes Methods for additional analyses, such as subgroup analyses and adjusted analyses
**Results**		
Participant flow (a diagram is strongly recommended)	13a 13b	For each group, the numbers of participants who were randomly assigned, received intended treatment, and were analyzed for the primary outcome For each group, losses and exclusions after randomization, together with reasons
Recruitment	14a 14b	Dates defining the periods of recruitment and follow-up Why the trial ended or was stopped
Baseline data	15	A table showing baseline demographic and clinical characteristics for each group
Numbers analyzed	16	For each group, number of participants (denominator) included in each analysis and whether the analysis was by original assigned groups
Outcomes and estimation	17a 17b	For each primary and secondary outcome, results for each group, and the estimated effect size and its precision (such as 95% confidence interval) For binary outcomes, presentation of both absolute and relative effect sizes is recommended
Ancillary analyses	18	Results of any other analyses performed, including subgroup analyses and adjusted analyses, distinguishing prespecified from exploratory
Harms	19	All important harms or unintended effects in each group (for specific guidance see CONSORT for harms)
**Discussion**		
Limitations	20	Trial limitations, addressing sources of potential bias, imprecision, and, if relevant, multiplicity of analyses
Generalizability	21	Generalizability (external validity, applicability) of the trial findings
Interpretation	22	Interpretation consistent with results, balancing benefits and harms, and considering other relevant evidence
**Other information**		
Registration	23	Registration number and name of trial registry
Protocol	24	Where the full trial protocol can be accessed, if available
Funding	25	Sources of funding and other support (such as supply of drugs), role of funders

*We strongly recommend reading this statement in conjunction with the CONSORT 2010 Explanation and Elaboration for important clarifications on all the items. If relevant, we also recommend reading CONSORT extensions for cluster randomized trials, non-inferiority and equivalence trials, non-pharmacological treatments, herbal interventions, and pragmatic trials. Additional extensions are forthcoming: for those and for up to date references relevant to this checklist, see www.consort-statement.org.

**Assessment of Potential Biases in Studies**

Two authors assessed RCTs using the Cochrane risk of bias tool.^9^

**Data Extraction and Data Sources**

One author extracted data from the included studies into extraction table including sample size, patients’ condition (acute/chronic TBI), outcome measures (favorable outcomes, mortality, and side-effect), citicoline’s dosage and route of use. While other authors checked accuracy and completeness of the extracted data.

**Analysis**

The outcomes were analyzed in two main groups for acute TBI management. Mortality and vegetative state (assessed in a single group) as well as good recovery and mild disability (as a favorable outcome) assessed with Glasgow Outcome Scale (GOS) after 3-6 months of patients' follow-up were considered primary outcomes. Severe disabilities were not included in this analysis. Occurrence of any citicoline adverse effects was analyzed as secondary outcome. For chronic TBI management, outcomes were analyzed for improvement in Neuropsychological state.

A p<0.05 was considered statistically significant through all statistical methods applied in this review. The effect sizes pooled in this meta-analysis included Risk Ratios (RR) and Standardized Mean Difference (SMD) along with their Confidence Interval (CI)=95%. Heterogeneity among the studies was assessed using I^2^ statistic and tested through and an I^2^ greater than 0.5 was considered for using random effects models.^10^

## Results

Articles related to citicoline intervention use for TBI management were published from 1991 to 2014.^11-14^ Zafonete et al.'s study was a big multicentric study a.k.a COBRIT (Citicoline Brain Injury Treatment) and halted in its 4th interim analysis due to non-significant outcome differences between placebo and intervention groups, but patients followed up to 180 days after injury, the data for 180-day results are included in this meta-analysis. Studies by Maldonado et al. and Shokouhi et al. were clinical trials comparing standard therapy with adjunct citicoline therapy. Both studies included patients with severe and moderate acute TBI (216 and 58 patients respectively). ^13,14^ Leon-Carrion et al.'s study was a small clinical trial of 10 patients for assessing nonrecognitive effects of citicoline. ^12^ COBRIT planned to enroll 1292 patients, which was halted in its 4th interim analysis with 1213 patients randomized in 2 placebo and citicoline groups; the primary outcome assessment on day 90 was available for 996 cases, while 180-day outcome enrolled 902 cases. ^11^ Table 1 summarized the characteristics of the included studies.

**Table 1:** Characteristics of Included Studies

Author (Year)	Sample Size(Type of Study)	Acute/Chronic TBI	Severity of Patients’ Condition	Intervention	Duration of Intervention (Follow up)	Outcome Assessment
**Leon-Carrion (2000)**	10(RCT intervention vs. placebo)	Chronic TBI	Patients with severe memory deficits	Oral 1 gram Daily	3 months in conjunction to neuropsychological treatment to both study groups(No further follow up after 3 months)	Before/After assessment of attention, vigilance, memory, verbal fluency and visoconstrictive abilities.
**Maldonado (1991)**	216(RCT Single-Blinded Case-Control)	Acute TBI	Patients with moderate or severe TBI	Intravenous (During admission) Days 1-2: 1 gram Q6hrs Days 3-4: 1 gram Q8hrs Then until discharge patients who wore phleoclisis received 1 gram Q8hr, and who didn’t received 1 gram Q12hrs Oral (After Discharge) 200mg Q8hr	The total duration of treatment was variable, depending on the evolution of the patient(3 months)	Comparing the evolution of patients who received conventional treatment with the evolution of those treated with citicoline using GOS.
**Shokouhi (2014)**	58 (RCT Double-Blinded Case-Control)	Acute TBI	Patients with moderate or severe TBI	Intravenous Citicoline (500 mg) Q6hr	15 Days(No further follow up after day 15)	1- Serum levels of fetuin-A and MGP: Days 6,12 2- Physical examination and GCS evaluation: Daily
**Zafonte (2012)**	1213 (RCT Phase-III)	Acute TBI	Patients with complicated mild, moderate or severe	Enteral or oral Citicoline (2000 mg) Daily	90 Days (Outcomes assessment in Days 30,90,180)	1- Functional and cognitive status assessed at day-90 using TBI-Clinical Trials Network Core Battery. 2- Days 30, 90,180 assessments for functional and cognitive improvement using GOS and long-term maintenance of treatment effects.

In total, this meta-analysis included 4 studies with 1196 patients, which COBRIT study comprised about 75% of the patients included in the analysis. The starting citicoline dose in these studies was 2 gr/day in COBRIT and Shokouhi et al.'s trials, 1 gr/day in Leon-Carrion et al.'s study and 4 gr/day in Maldonado et al. (reduced to 3 gr/day after day 3-4 of intervention and 2 gr/day in case phlebitis was recognized).

A parallel study to COBRIT showed an overall improvement process of psychiatric characteristics in TBI patients over 180 days of assessment, with better outcomes on days 30-90. Better outcomes were observed for female participants in comparison to the males. Hispanic had better improvement and African-Americans had less improvement process when compared to the Whites as the ethnic/race’s analysis reference group.^15^ There was also a study by Salehpour et al. that has reported neuroprotective effect of Citicoline on the reduction of serum levels of MalonDiAldehyde (MDA) as a marker of oxidative stress, but the study was excluded from our review because no clinical outcome was reported.^16^

**Fig 2:**
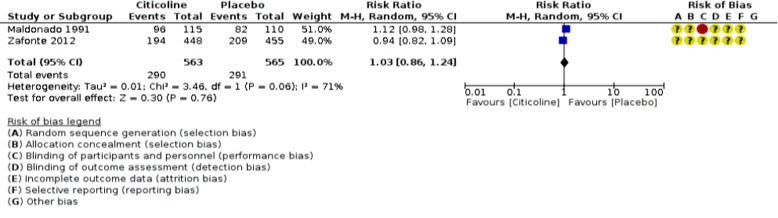
Citicoline favorable outcome- GOS results

**Fig 3:**
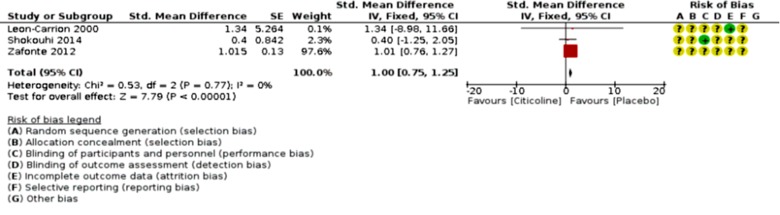
Citicoline favorable outcomes (at all neuropsychological)

**Fig 4:**
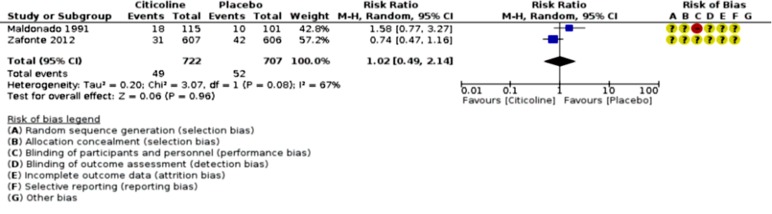
Citicoline mortalities

**Fig 5:**
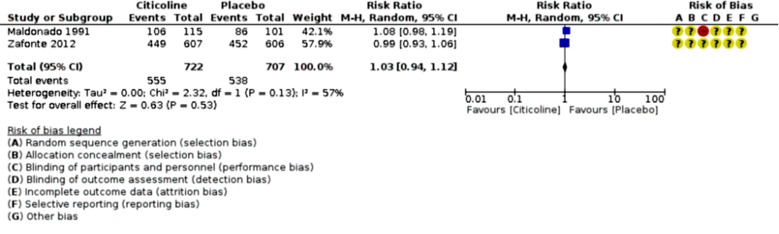
Citicoline side-effects

An analysis of outcomes showed no significant change in GOS outcome (p=0.76; RR 1.03, 95% CI 0.86 to 1.24; participants = 1128; studies = 2; I2 = 71%, Figure 2), but significant favorable Neuro-cognitive changes in placebo-control group despite studies’ heterogeneity (p <0.00001; SMD 1.00, 95% CI 0.75 to 1.25; participants = 971; studies = 3, Figure 3). However, the comparison of COBRIT study’s days-90 and 180 GOS outcomes demonstrated improvements in day 180 outcomes. ^11^ With respect to mortality and vegetative-state outcomes, only two studies by Maldonado et al. and COBRIT trials have reported these outcomes that were analyzed as a joint outcome in the present review (p=0.96; RR 1.02, 95% CI 0.49 to 2.14; participants = 1429; studies = 2; I2 = 67%, Figure 4). No significant difference was detected in side-effects of the intervention between trial groups (p=053; RR 1.03, 95% CI 0.94 to 1.12; participants = 1429; studies = 2; I2 = 57%, Figure 5).

## Discussion

COBRIT study for citicoline use in TBI management seems to be like CRASH, SYNAPSE and ProTECT-III or EPO-TBI in their own fields of interest,^4,11,17-19^as it was a big multi-centric placebo-control RCT of citicoline. Its halt in 4th interim analysis may resulted in less participation of patients during follow-up process, but it was not found to have a significant difference between groups. Overall assessment of outcomes didn’t demonstrate any significant effect of citicoline favorable outcomes especially in GOS. However, assessment of GOS for days 90 and 180, improvements were slightly better but not significant in COBRIT study (from P=0.97 to P=0.43). There is significant improvement of placebo-control group patients in neurocognitive state rather than intervention group. Neither mortality nor side-effects of citicoline versus control groups were significantly different.

Maldonado et al.'s was the 2nd largest study after COBRIT in these search results. This study along with the studies by Leon-Carrion et al. and Shokouhi et al. had supported a beneficence in citicoline use for severe and moderate TBIs. Nevertheless, it was questioned by CO-BRIT that didn’t report such an effect in outcomes neither in day-90 nor day-180.^11-14^ Also, a significant better outcome change was obvious in mildly complicated cases on day-180 outcome in COBRIT study.^11^ Considering the large weight of COBRIT study, the variations of citicoline doses and outcome assessments reported by other three studies could easily be suppressed by COBRIT results. However, current use of citicoline for TBI in acute or chronic phase is no more recommended by the results of this review. Nevertheless, neuro-cognitive benefits of citicoline need to be investigated through further research for chronic TBI patients.

Improvement in psychiatric assessments of TBI patient after an assault differs among individuals. There should be supportive Psychological First Aid (PFA) tools for primary survivors of the assault. The Johns Hopkins University’s course of PFA-RAPID which stands for Rapport and Reflective Listening, Assessment of needs, Prioritization, Intervention, and Disposition is available at https://www.coursera.org/course/psychfirstaid to guide the triage and primary effective intervention of healthcare providers for trauma assaults survivors, as further than the insult, sub-acute complications during recovery of patients especially in two-third of severe impaired TBI patients^20^ which may affect their families’ lives, too.

## Conclusion

The available evidence doesn’t support current routine use of Citicoline for acute TBI management. Citicoline use for managing impaired neuro-cognitive conditions in chronic TBI patients is weak and needs further research.
